# Mapping the Hypoxic Fitness Landscape of Retinal Pigment Epithelial Cells

**DOI:** 10.3390/ijms27062857

**Published:** 2026-03-21

**Authors:** Ozlem Calbay, Chen-Lin Hsieh, Charles Lu, Sujana Ghosh, Vinny Vijaykumar, Isabella Watts, Harry Sweigard, Jarel Gandhi, Anneke I. den Hollander

**Affiliations:** 1 Quantitative Medicine and Genomics, Research & Development, AbbVie, North Chicago, IL 60064, USA; channing0123@gmail.com (C.-L.H.); charles.lu@abbvie.com (C.L.); sujana.ghosh@abbvie.com (S.G.); vinny.vijaykumar@abbvie.com (V.V.); isabella.watts@abbvie.com (I.W.); jarel.gandhi@abbvie.com (J.G.);; 2 Ophthalmology Discovery, Research & Development, AbbVie, Irvine, CA 92618, USA; harry.sweigard@abbvie.com

**Keywords:** hypoxia, low oxygen, retinal pigment epithelium, RPE, CRISPR/Cas9, cell fitness, age-related macular degeneration, AMD

## Abstract

Chronic hypoxia is a hallmark of aging and retinal diseases such as age-related macular degeneration (AMD), yet the molecular mechanisms that enable retinal pigment epithelium (RPE) cells to survive under sustained low-oxygen conditions remain poorly understood. To address this, we conducted transcriptomic profiling and a genome-wide CRISPR-Cas9 loss-of-function screen in ARPE-19 cells exposed to chronic hypoxia (1% and 5% O_2_), mimicking the retinal disease environment. The CRISPR screen identified genes whose loss compromises RPE viability or fitness under hypoxia, while transcriptomic profiling revealed oxygen-dependent shifts in key functional modules. These findings converged on pathways related to mitochondrial function, extracellular matrix remodeling, vascular signaling, and cell cycle regulation, identifying unique functional nodes specific to RPE cells. These core processes are also implicated in retinal diseases, such as AMD. Together, these complementary approaches provide an integrated view of the molecular networks driving RPE adaptation to hypoxic stress and highlight novel gene candidates that may serve as therapeutic targets in retinal disease.

## 1. Introduction

The retinal pigment epithelium (RPE) is a metabolically active, post-mitotic monolayer essential for maintaining photoreceptor function and overall visual integrity. It plays a central role in retinal homeostasis by transporting oxygen and nutrients from the choroid, phagocytosing photoreceptor outer segments, regulating ion balance, and facilitating waste clearance [1]. With aging and disease progression, structural and functional impairments including RPE dysfunction, Bruch’s membrane thickening, and drusen accumulation disrupt the exchange of nutrients and gases, creating a state of relative hypoxia in the retina [2]. In response to declining oxygen levels, RPE cells initiate adaptive stress responses, such as the upregulation of *vascular endothelial growth factor* (*VEGF*), which can drive pathological neovascularization and accelerate retinal degeneration [2,3].

Hypoxia is a key pathological feature in a range of retinal diseases, including age-related macular degeneration (AMD), diabetic retinopathy, and retinal vein occlusion [4,5]. While acute hypoxic responses, particularly those mediated by *hypoxia-inducible factors* (*HIFs*), are well characterized, the long-term, graded, and context-specific adaptations of RPE cells to sustained hypoxia remain poorly understood. Recent studies revealed that hypoxia can drive profound metabolic reprogramming in RPE cells, including disruptions in glucose and lipid metabolism, which ultimately compromises photoreceptor support and viability [4]. Additionally, hypoxia has been shown to promote ferroptotic cell death in the RPE by enhancing Fenton chemistry and oxidative stress, underscoring the relevance of iron metabolism in RPE degeneration [6]. Notably, hypoxia also modulates the dual roles of *HIF-1α* in promoting protective metabolic adaptation in dry AMD and pathological *VEGF*-driven angiogenesis in wet AMD, reflecting the complex, disease-specific impact of hypoxic signaling [7].

The hypoxic responses of the RPE involve a spectrum of molecular changes, including transcriptional reprogramming, metabolic shifts, epigenetic remodeling, and activation of autophagy. *BNIP3*-mediated autophagy, for instance, has been shown to confer protection to RPE cells under hypoxic stress [8], while early studies demonstrated that RPE viability is differentially affected by the severity and duration of hypoxia, with higher oxygen deprivation inducing apoptosis that could be mitigated by antioxidant treatment [9]. Despite these advances, a comprehensive understanding of how different levels and durations of hypoxia influence gene regulatory networks, survival pathways, and stress responses in RPE cells is still lacking.

Here, we address this gap by systematically mapping the hypoxic fitness landscape of ARPE-19 cells through the integration of time-resolved RNA sequencing and genome-wide CRISPR-Cas9 screening under prolonged hypoxic conditions (1% and 5% O_2_). This combinatorial approach allows us to delineate transcriptional programs specific to mild (5%) and severe (1%) hypoxia, identify disease-relevant pathways implicated in AMD and other eye diseases, and uncover key genetic dependencies required for RPE survival and function under severe oxygen deprivation. By intersecting transcriptomic and functional genomic datasets, we define the molecular circuits that orchestrate the response of the RPE to chronic hypoxia, providing new mechanistic insights into hypoxia-driven retinal degeneration and potential targets for therapeutic intervention.

## 2. Results

### 2.1. Distinct Molecular Responses in RPE Cells Elicited by Varying Degrees of Hypoxia

**Figure 1:**
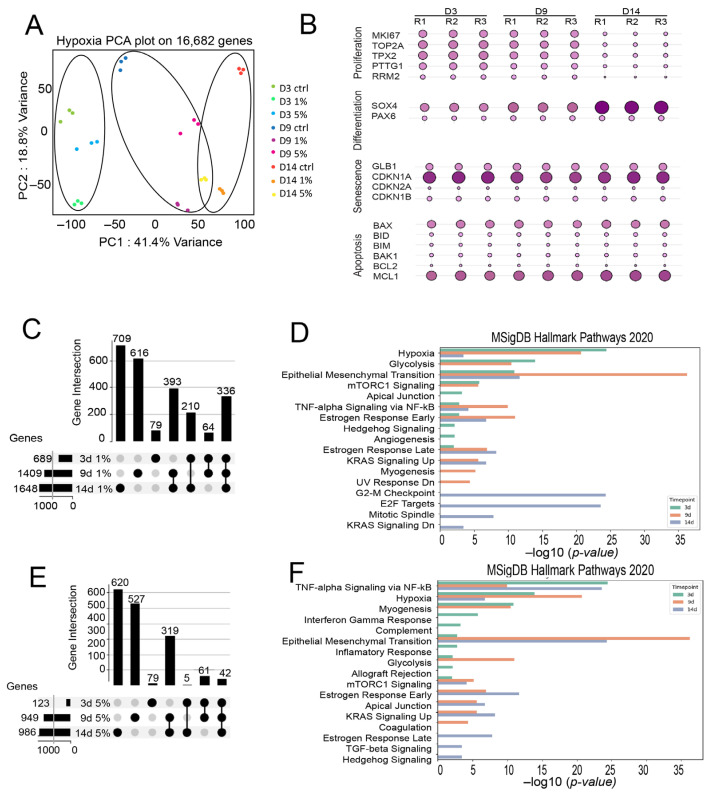
Temporal Dynamics of Gene Expression and Pathway Enrichment. (**A**) PCA of RNA-seq data from ARPE-19 cells cultured under 1%, 5%, and 21% O_2_ for 3, 9, and 14 days. PCA was performed on 16,582 genes to assess sample-level variability. PC1 (41.4%) separates samples primarily by hypoxia duration, while PC2 (18.8%) captures differences driven by oxygen concentration. Ellipses represent sample clustering by timepoint, highlighting distinct transcriptional programs induced by acute versus chronic hypoxia exposure. (**B**) Dot plot showing expression levels (TPM) of selected normoxia-associated genes involved in proliferation, differentiation, senescence and apoptosis regulation across all time points and oxygen conditions. Dot size corresponds to raw expression values, and color intensity reflects transcript abundance (TPM), illustrating temporal and oxygen-dependent modulation of key biological processes under hypoxic stress. (**C**,**E**): Upset plots illustrating the intersection of differentially expressed genes (DEGs) at 3, 9, and 14 days. Bars with three connected dots represent genes shared across all timepoints, whereas bars with two dots represent genes shared exclusively between those two timepoints and not the third. A persistent core gene set therefore yields a larger triple intersection than pairwise overlaps. Using a cutoff of FDR-adjusted *p*-value < 0.05 and |log2 fold change| > 1, we identified 689 and 1648 DEGs at 3 days under 1% and 5% O_2_; 948 and 1409 DEGs at 9 days under 1% and 5% O_2_; and 986 and 1067 DEGs at 14 days under 1% and 5% O_2_, respectively. Horizontal bars represent the total number of DEGs at each time point, while vertical bars indicate the number of shared or unique genes across different time points. Dots below the bars denote the specific time point combinations contributing to each intersection. The top upset plot represents one dataset, while the bottom plot shows another comparison, highlighting differences in gene expression dynamics over time. (**D**,**F**). Hallmark Pathway Enrichment Analysis of RPE mRNA Expression Profiles Over Time, 1% and 5%, respectively. Bar plot depicting the enrichment scores (−log10(*p*-value)) for selected Hallmark 2020 gene sets from MSigDB in mRNA expression profiles at three different time points: 3 days (green), 9 days (salmon), and 14 days (purple). Higher −log10(*p*-values) indicates more statistically significant enrichment of the corresponding pathway.

To investigate the transcriptional response of RPE cells to hypoxia, we performed RNA sequencing (RNA-seq) on human ARPE-19 cells (CRL-2302; ATCC, Manassas, VA, USA) exposed to normoxia (21% O_2_) and hypoxia (1% and 5% O_2_) for 3, 9, and 14 days. RNA-seq was performed after 14 days of hypoxic exposure to capture stabilized transcriptional responses following early adaptation to low oxygen. Hypoxia-induced gene expression changes are known to occur rapidly and frequently plateau over time, making intermediate timepoints suitable for profiling sustained transcriptional programs. Principal component analysis (PCA) plots of RNA-seq data showed minimal batch effects and sample variation, while clustering patterns were influenced by both time and oxygen conditions (Figure 1A). Differentially expressed genes (DEGs) were identified by comparing each hypoxia treatment condition to its corresponding normoxia control.

First, we characterized the cellular state under normoxic conditions at 3, 9 and 14 days to assess long-term culture effects. We examined well-established markers of proliferation (*MKI67*, *TOP2A*, *TPX2*, *PTTG1*, *RRM2*), differentiation (*SOX4*, *PAX6*) [10], senescence (*GLB1*, *CDKN1A*, *CDKN2A*, *CDKN1B*) [11], and apoptosis (*BAX*, *BID*, *BIM*, *BAK1*, *BCL2*, *MCL1*) [12]. Our analysis revealed a downregulation of proliferation markers and an upregulation of differentiation markers over time, suggesting a shift toward a more differentiated epithelial phenotype. Meanwhile, the absence of significant changes in senescence and apoptotic markers indicates that the cells remain viable during long-term culture (Figure 1B).

Next, we examined the transcriptomic profiles of cells exposed to 1% and 5% hypoxia across multiple time points to capture the temporal dynamics of gene expression in response to sustained hypoxic stress. This time-resolved analysis enabled us to uncover how cellular transcriptional programs evolve over time under varying degrees of oxygen deprivation. Upset plot analysis (Figure 1C,E) showed a progressive shift in gene expression, with a significant number of unique DEGs at each time point, indicating distinct transcriptional programs. While a core set of genes remained consistent, the highest overlap occurred between 9 and 14 days, suggesting a gradual regulatory transition in later stages. We employed Hallmark gene set enrichment analysis (MSigDB 2020) [13] to characterize the hypoxia-induced pathways in our transcriptomic datasets (Figure 1D,F). Comparative analysis revealed distinct temporal transcriptional responses between 1% and 5% oxygen conditions. Initially, both conditions induced classical hypoxia-responsive genes, but the magnitude and specificity of regulation varied over time.

At 1% hypoxia, ARPE-19 cells rapidly activated hypoxia, glycolysis, and EMT pathways by day 3, indicating early metabolic and phenotypic changes (Figure 1D). Concurrent *mTORC1*, apical junction, and estrogen response signaling suggested nutrient sensing and remodeling. By day 9, these core pathways persisted, with intensified *TNF-α/NF-κB* and *KRAS* signaling reflecting ongoing stress responses and adaptation. At day 14, the transcriptome shifted toward proliferation and transformation, marked by enrichment of *E2F* Targets, G2-M Checkpoint, and mitotic spindle pathways, alongside chronic stress and hormonal signaling changes.

Under 5% hypoxia, ARPE-19 cells showed an early inflammatory and immune-like response by day 3, marked by *TNF-α/NF-κB*, hypoxia, interferon gamma, and complement pathway activation, reflecting an acute inflammatory and stress reaction (Figure 1F). By day 9, hypoxic and inflammatory signaling persisted alongside metabolic and structural remodeling. At day 14, the enrichment of *KRAS*, *TGF-β*, and Hedgehog signaling indicated sustained inflammation and a shift toward regenerative or stem-like traits.

Despite differences, both 1% and 5% hypoxia shared core features such as sustained Hypoxia, EMT, and *TNF-α/NF-κB* signaling, reflecting conserved stress and plasticity responses. By day 9, the common enrichment of glycolysis, *mTORC1*, and estrogen pathways indicated similar metabolic adaptation. On day 14, both showed EMT and *KRAS* activation, suggesting long-term survival shifts. However, 5% hypoxia triggered early immune-like responses (e.g., interferon gamma, complement), while 1% hypoxia uniquely activated cell cycle and DNA damage pathways later, indicating a more transformation-prone profile. Therefore, our findings underscore that the hypoxic level and duration critically determine the outcome of transcriptomic networks and signaling pathways.

### 2.2. Severe Hypoxia (1%) Induces Molecular Responses Enriched for AMD and Ocular Disease-Associated Biological Functions in RPE Cells

**Figure 2:**
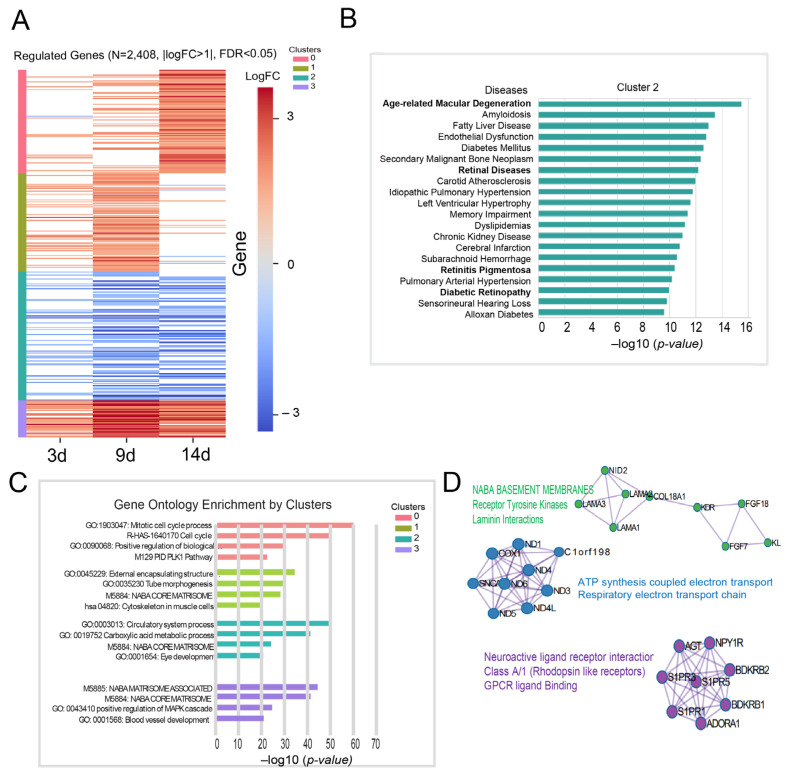
Identification of hypoxia-responsive transcriptional modules and their association with retinal disease pathways. (**A**) K-means clustering of hypoxia-responsive genes based on RNA-seq data. Unsupervised k-means clustering was performed on filtered differentially expressed genes (FDR < 0.05, |log_2_FC| > 1) in 1% hypoxic condition across timepoints in ARPE-19 cells. The optimal number of clusters was determined using the elbow and silhouette methods. Heatmap displays logFC for each cluster, highlighting distinct temporal and oxygen-dependent gene expression programs. (**B**) Disease enrichment analysis of hypoxia-induced gene clusters. Genes from each k-means cluster were analyzed for enrichment in disease-associated gene sets using the DisGeNET database. Cluster 2, in particular, showed significant enrichment for genes implicated in age-related macular degeneration (AMD) and other retinal degenerative diseases, suggesting activation of disease-relevant pathways under hypoxia. (**C**) Gene Ontology (GO) enrichment analysis by cluster. GO Biological Process enrichment was performed for each cluster using IPA. Top enriched pathways per cluster are shown, revealing functional specialization of transcriptional modules. (**D**) MCODE-based network analysis reveals functional hubs within hypoxia-responsive genes. Protein–protein interaction networks were constructed from the differentially expressed genes, and the MCODE algorithm was applied to identify densely connected modules. Each MCODE cluster was subjected to pathway enrichment analysis, highlighting key functional hubs such as mitochondrial metabolism, chromatin remodeling, cell cycle control, and stress signaling—processes central to retinal cell adaptation under chronic hypoxia.

To identify biological functions associated with 1% hypoxia, given that this condition exhibited the greatest viability-associated alterations in transcriptomic profiles during prolonged hypoxic exposure, and to uncover disease-relevant molecular responses to hypoxic stress, we applied k-means clustering to the gene expression data (Figure 2A). This approach effectively partitioned the data, revealing four distinct temporal gene clusters. Cluster 0 showed a strong enrichment at day 14, cluster 1 peaked on day 9, cluster 2 progressively decreased over time, and cluster 3 remained consistently enriched across all time points. These clusters enabled the delineation of hypoxia-induced regulatory networks and their connection to RPE fitness gene modules under sustained low oxygen conditions.

To elucidate the functional differences among these gene clusters and identify the most significant biological pathways and disease associations, we performed enrichment analyses. Each cluster displayed unique biological themes (Figure 2B,C). Cluster 0 was enriched for cell cycle regulation, implicating chronic hypoxia in cell proliferation control. Cluster 1 showed enrichment in extracellular matrix (ECM) organization and tissue formation, suggesting cellular remodeling as an adaptive response to hypoxia. Cluster 2 was significantly associated with vascular development, circulatory processes, metabolic activity, and cell proliferation, pointing to roles in tissue homeostasis and remodeling. Notably, this cluster also showed strong enrichment for AMD and other retinal diseases (Figure 2B, Supplementary Figure S1A), suggesting a compelling transcriptional link to ocular disease mechanisms. Cluster 3 was enriched for ECM signaling, differentiation, and disease-related signaling pathways, reflecting a broader stress-responsive regulatory network.

To further investigate functional modules potentially relevant to AMD and other ocular pathologies within our hypoxia-responsive gene set, we conducted protein–protein interaction (PPI) enrichment analysis on the genes in Cluster 2. This revealed 17 densely connected components using the MCODE algorithm (Figure 2D, Supplementary Figure S1B). Subsequent Gene Ontology and pathway enrichment analyses identified several modules with strong functional relevance.

One module showed strong enrichment for biological processes related to ATP synthesis-coupled electron transport (log10(*p*-value) = −19.3), mitochondrial ATP synthesis (log10(*p*-value) = −19.3), and the respiratory electron transport chain (log10(*p*-value) = −18.3). This module contained several mitochondrial-encoded genes, including *MT-CO1*, *ND1*, *ND3*, *ND4*, *ND4L*, *ND5*, and *ND6*, which encode core subunits of Complex I and Complex IV of the electron transport chain. Additional genes in the module included *SNCA* and *C1orf198*, both associated with mitochondrial function. These results demonstrate a coordinated transcriptional repression of mitochondrial electron transport components, particularly those encoded by the mitochondrial genome, suggesting a shift from oxidative phosphorylation (OXPHOS) to glycolysis as an adaptive response [14,15].

Two other modules contained genes showing significant associations with “Naba Basement Membranes” (log10(*p*-value) = −12.4), “Signaling by Receptor Tyrosine Kinases” (log10(*p*-value) = −11.4), and “Laminin Interactions” (log10(*p*-value) = −10.4). *NID2*, *LAMA1*, *LAMA2, LAMA3*, and *COL18A1* encode critical structural components of the basement membrane and are essential for maintaining epithelial polarity and cell adhesion [16,17]. *KDR* (*VEGFR2*), *FGF7*, *FGF18*, and KL, genes involved in receptor tyrosine kinase-mediated growth factor signaling, contribute to epithelial repair, proliferation, cell survival, oxidative stress resistance, and angiogenic regulation [18,19]. These findings suggest that severe hypoxia induces transcriptional programs leading to a loss of basement membrane structural integrity, disruption of epithelial architecture, and impaired growth factor-mediated signaling, ultimately disturbing tissue homeostasis and cellular resilience.

Another module demonstrated strong enrichment for signaling pathways, including “Neuroactive ligand-receptor interaction” (log10(*p*-value) = −15.3), “Class A/1 (Rhodopsin-like receptors)” (log10(*p*-value) = −13.0), and “*GPCR* ligand binding” (log10(*p*-value) = −11.9). This module comprises several G protein-coupled receptors (GPCRs), including *AGT*, *NPY1R*, *S1PR1*, *S1PR3*, *S1PR5*, *BDKRB1*, *BDKRB2*, and *ADORA1*, which coordinate vasoregulatory, neuropeptidergic, and lipid-mediated signaling pathways essential for maintaining tissue homeostasis, vascular tone, and cellular responses to stress [20]. This coordinated and prolonged transcriptional suppression of GPCR pathways suggests potential impairment of neurovascular coupling, angiostasis, and barrier maintenance.

### 2.3. Oxygen-Dependent Genetic Determinants of ARPE-19 Cell Fitness Revealed by Genome-Wide CRISPR Screening

#### 2.3.1. Genome-Wide CRISPR Screen Using a Pooled sgRNA Library in RPE Cells

**Figure 3:**
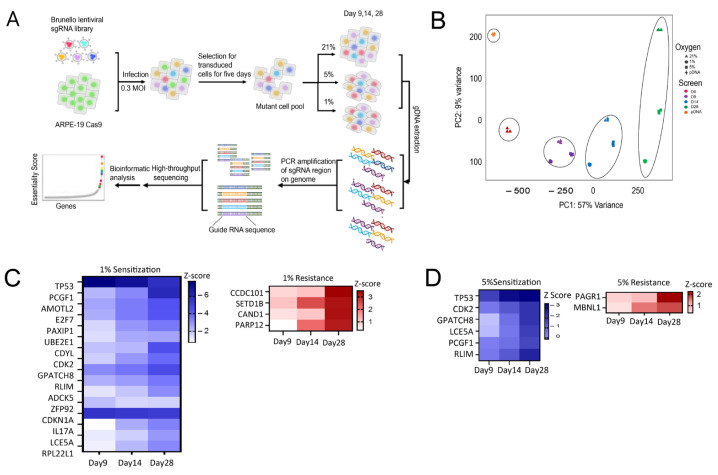
Genome-wide CRISPR-Cas9 screen identifies essential regulators of RPE cell survival under hypoxic stress. (**A**) Schematic of the genome-wide CRISPR-Cas9 screening workflow. ARPE-19 cells stably expressing Cas9 were transduced with the Brunello genome-wide sgRNA library and selected under puromycin. Cells were exposed to chronic hypoxia (1% or 5% O_2_) or maintained under normoxia (21% O_2_) for 9, 14 and 28 days. Genomic DNA was harvested at endpoint, and sgRNA abundance was quantified by next-generation sequencing. Gene-level essentiality scores were computed using custom script to identify hypoxia-specific viability regulators. (**B**) PCA of CRISPR screen replicates. The shape of each data point represents oxygen level, while the color indicates duration of exposure to that condition. Treatment groups separate clearly in principal component space; ovals are included to visually group samples by time point, highlighting temporal progression within each oxygen condition. sgRNA abundance profiles show distinct clustering of hypoxic (1% and 5% O_2_) versus normoxic samples, indicating reproducible and condition-specific selective pressures. Biological replicates cluster tightly, demonstrating screen robustness and strong hypoxia-driven divergence in genetic dependencies. (**C**) Top genes conferring sensitivity or resistance under 1% O_2_. (**C**) Top genes conferring sensitivity or resistance under 1% O_2_. The top hits (FDR < 0.1) are visualized in a heatmap showing Z-score normalized gene-level fold changes across all time points. Sensitivity genes are depleted under hypoxia, indicating their loss impairs adaptation and survival. Resistance genes are enriched, suggesting their loss may promote survival by removing barriers to hypoxic adaptation or cell growth. (**D**) Top genes conferring sensitivity or resistance under 5% O_2_. Similar to (**C**), heatmap displays genes essential or dispensable under 5% hypoxia.

Pooled genome-wide CRISPR screens were conducted using the Brunello lentiviral sgRNA library (Broad Institute, Cambridge, MA, USA), which targets around 20,000 genes with 76,441 guide RNAs (gRNAs) [21], in a Cas9-expressing ARPE-19 cell line. The cell line exhibited stable and high Cas9 activity in *EGFP* knockout (KO) reporter assays (Supplementary Figure S2A). Overall experimental design is summarized in Figure 3A. After puromycin (Gibco; Thermo Fisher Scientific, Waltham, MA, USA) selection for gRNA expression, cells were cultured under three distinct oxygen tensions to identify key regulators that exhibit differential fitness as a function of oxygen tension [22]. To maintain optimal conditions for cell growth across varying oxygen levels, all experiments were conducted in 5-chamber CellSTACK flasks (Corning Inc., Corning, NY, USA), providing ample surface area. Cell doubling times were consistent under different oxygen tensions: 24 h at both 21% and 5% O_2_, and 36 h at 1% O_2_. These conditions ensured that cell growth and proliferation remained stable throughout the experiment, despite fluctuations in oxygen availability. Samples of the genome-wide screen were collected at 9, 14, and 28 days post-infection to assess changes in gene knockout abundance. The screening was conducted over a 28-day hypoxic exposure period. Dropout-based screens require extended culture corresponding to approximately 12–14 population doublings to enable selective depletion of cells lacking genes essential for long-term survival under hypoxic conditions.

Before applying statistical tests to identify significant hits, we implemented quality control (QC) measures to ensure data reliability. We assessed data quality and confirmed proper library representation (Supplementary Figure S2B), with most gRNAs distributed within the 1–100 counts per million (CPM) range, indicating strong coverage. One sample at day 14 5% O_2_ was excluded due to low quality. Gini coefficients (Supplementary Figure S2C) confirmed even gRNA representation at baseline (pDNA, Day 0: ~0.3), with increasing skewness over time (Day 9: 0.4–0.5, Day 14: 0.5–0.55, Day 28: 0.6–0.7), reflecting selective pressure. PCA (Figure 3B) demonstrated a time-dependent shift in cell populations during the CRISPR screen. Plasmid DNA (pDNA) of the original guide RNA library was sequenced to assess library quality and guide RNA representation, serving as a baseline reference for comparison with Day 0 pre-selection samples. PCA showed pDNA and Day 0 clustering distinctly, with a transition observed by Day 9 and stabilization by Day 28, indicating strong selection effects. Collectively, the data generated provides a robust foundation for further analysis.

#### 2.3.2. Identification of Oxygen-Dependent Genetic Regulators in ARPE-19 That Impact Cellular Fitness Across Oxygen Conditions

To uncover oxygen-dependent genetic regulators influencing cellular fitness under hypoxia, we analyzed gene KOs showing differential abundance between 21% O_2_ and 1% or 5% O_2_ at 28 days, yielding critical insights into hypoxia-related biological processes. An FDR cutoff of 0.1 was applied to control for multiple comparisons and reduce the likelihood of false positives. We examined gRNA depletion and enrichment of the top-hit genes (Figure 3C), where depleted genes showed reduced gRNA abundance, indicating impaired survival under hypoxia (sensitivity genes), while enriched genes had overrepresented gRNAs, suggesting that their knockout conferred a survival advantage in hypoxic conditions (resistance genes). This approach allowed us to systematically identify genes whose knockout altered cellular survival in hypoxic conditions.

Under 1% O_2_ hypoxia, 15 sensitivity genes were identified, including *TP53* (cell cycle arrest and apoptosis), *PCGF1* (chromatin remodeling), *AMOTL2* (cellular junction integrity), and *E2F7* (hypoxic cell cycle adaptation). Other notable genes included *PAXIP1* (DNA repair), *UBE2E1* (protein degradation), *CDYL* (transcriptional repression), *GPATCH8* (RNA processing), *RLIM* (proteasomal regulation), and *CDKN1A* (cell cycle arrest mediator). Additionally, genes such as *ADCK5*, *ZFP92*, *IL17A*, *LCE5A*, and *RPL22L1* are involved in mitochondrial function, transcriptional regulation, inflammation, and epithelial barrier maintenance. Four genes conferred resistance to severe hypoxia, including *CCDC101* and *SETD1B* (chromatin dynamics regulators), and *PARP12* (apoptotic signaling attenuation). Under 5% O_2_ hypoxia, six sensitivity genes were identified, with significant overlap with the 1% O_2_ condition, including *TP53*, *CDK2*, *PCGF1*, *GPATCH8*, *RLIM*, and *LCE5A*, reinforcing their roles in adaptation to hypoxia. Two resistance genes, *PAGR1* and *MBNL1*, were identified in 5% O_2_, associated with chromatin-associated stress adaptation and alternative splicing. These findings emphasize the differential gene dependencies in response to varying levels of oxygen deprivation and highlight genes that confer susceptibility or resistance to hypoxia in the prolonged hypoxia readout at 28 days.

### 2.4. High-Fidelity CRISPR Screening Identifies Genes Critically Required for Survival Under Hypoxia in ARPE-19 Cells

#### 2.4.1. Validation of CRISPR Screen by Essential Gene Depletion Analysis

**Figure 4:**
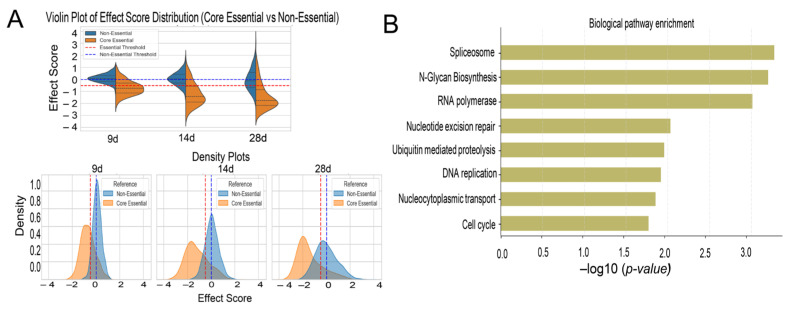
Comparative Analysis of Gene Essentiality and Pathway Enrichment. (**A**). Split violin plots comparing essential and non-essential genes across time points (upper panels) reveal clear differences in their depletion patterns under hypoxia. Split violin plots display the distribution of median gene-level depletion scores under 1% O_2_ conditions, highlighting a significant loss of viability for core essential genes compared to non-essential gene controls. These differences are further illustrated by density versus effect size plots, demonstrating a pronounced shift between essential and non-essential gene populations over time, reflecting their distinct functional roles during hypoxic adaptation. (**B**). Pathway enrichment analysis of essential genes. Top biological pathways enriched among genes identified as essential under hypoxic stress (FDR < 0.1), as determined by gene set enrichment analysis (GSEA) and GO Biological Process categories. Key functional hubs include cell cycle regulation, DNA damage response, mitochondrial maintenance, and ubiquitin-mediated proteolysis.

To validate our CRISPR screen, we compared gene KO effect scores against established essential and non-essential gene datasets from [23]. We analyzed KO data under normoxic conditions (21% O_2_) at 14 and 28 days, using Day 0 as a baseline. Genes were classified as essential (median effect score ≤ −0.5) or non-essential (median effect score > 0), ensuring robust assessment of gene essentiality by minimizing the impact of outliers. Our analysis demonstrated a clear separation in effect score distribution (Figure 4A, Supplementary Figure S2D), with essential genes exhibiting significantly lower median scores than non-essential genes. This confirms that targeting essential genes consistently impaired cell viability, while targeting non-essential genes had minimal impact. Pathway enrichment analysis of depleted genes further supported these findings, revealing strong enrichment for fundamental biological processes, including, mRNA splicing, DNA replication, proteasomal degradation, and mitotic progression (Figure 4B). These results align with known gene essentiality networks [24], validating the biological relevance of our identified hits.

#### 2.4.2. Secondary Validation via Individual Gene Knockouts

**Figure 5:**
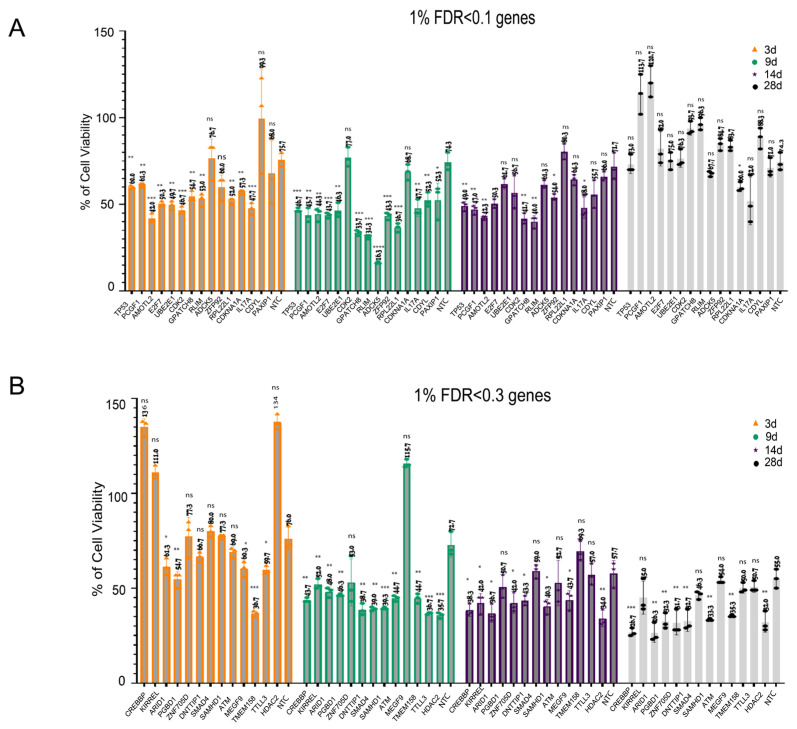
Functional validation of hypoxia-essential genes identified by CRISPR screening. (**A**). Functional validation of top essential genes (FDR < 0.1). Bar plots showing viability-based confirmation of CRISPR knockouts in ARPE-19 cells under hypoxia (1% O_2_). Knockout of selected genes significantly reduced cell viability relative to control sgRNAs, confirming their essential roles under metabolic stress. (**B**). Validation of additional hypoxia-essential genes (FDR < 0.3). Extended validation panel of genes meeting a relaxed significance threshold (FDR < 0.3). Knockout of these genes also resulted in viability loss, supporting their potential roles in hypoxia adaptation and stress tolerance. The viability effect of each gene at each time point was compared to the corresponding non-targeting control using an unpaired two-tailed Student *t*-test (*n* = 3 biological replicates per group). Statistical significance is indicated as follows: ns: not significant, * *p* < 0.05, ** *p* < 0.01, and *** *p* < 0.001, **** *p* < 0.0001.

To rigorously validate the top hits from our genome-wide CRISPR viability screen, we selected a subset of genes for individual knockout experiments using the top two individual sgRNAs identified in our screen. We then performed a longitudinal analysis of cell viability at 3, 9, 14, and 28 days using the CellTiter-Glo Luminescent Cell Viability Assay (Promega, Madison, WI, USA) (Figure 5A). This subset includes the top 15 hits from 1% hypoxia, all of which also overlap with the hits from 5% hypoxia (FDR < 0.1): *TP53*, *PCGF1*, *AMOTL2*, *E2F7*, *UBE2E1*, *CDK2*, *GPATCH8*, *RLIM*, *ADCK5*, *ZFP92*, *RPL22L1*, *CDKN1A*, *IL17A*, *CDYL*, and *PAXIP1*. Additionally, we included 12 genes with FDR < 0.3 (Figure 5B, Supplementary Figure S3A) in the CRISPR screen: *ATM*, *DNTTP1*, *KIRREL*, *MEGF9*, *PGBD1*, *SMAD4*, *TMEM158*, *SAMHD1*, *CREBBP*, *ZNF705D*, *HDAC2*, and *TTLL3*. Each gene was tracked throughout the entire 28-day period under both hypoxic conditions (1 and 5%), offering a detailed perspective on how its knockout impacted cell viability over time. Some of these genes also exhibited essentiality under 5% hypoxia (Supplementary Figure S3B,C). While some genes were not initially identified as sensitivity hits under 5% hypoxia in our CRISPR screens, we continued to assess them under 5% hypoxia to ensure a comprehensive, time-resolved understanding of gene KO effect.

At 3 days, significant reductions in cell viability were observed across multiple knockout conditions. Specifically, we noted substantial reductions for *TP53*, *PCGF1*, *AMOTL2*, *E2F7*, *UBE2E1*, *CDK2*, *GPATCH8*, *RLIM*, *ZFP92*, *RPL22L1*, *CDKN1A*, and *IL17A* as 0.1 FDR top hits and *ARID1*, *PGBD1*, *MEGF9*, *TMEM158*, and *TTLL3* for the 0.3 FDR top hits. This demonstrates the critical role of these genes in early cellular adaptation to hypoxia. By 9 days, the trend persisted for the majority of the hits and viability further dropped as low as 16.3 for *ADCK5*, 33.7 for *GPATCH8*, and 31.3 for *RLIM*, with sustained viability reductions observed for the same gene sets. Notably, *TP53* and *CREBBP* knockouts exhibited dramatic reductions in viability over time, highlighting their importance in maintaining cell survival under prolonged hypoxic stress. The non-targeting control consistently showed relatively high viability, confirming that the observed effects were specific to the gene knockouts. At 14 and 28 days, the knockout lines continued to exhibit significantly reduced viability compared to the non-targeting control, confirming the sustained impact of these gene knockouts on cell survival under chronic hypoxia. While an increase in viability was observed for some genes at 28 days, the overall trend confirmed the temporal impact of these gene knockouts on cell survival under chronic hypoxia. Taken together, some genes caused a progressive viability decline (e.g., sustained sensitivity), others led to an initial dip followed by recovery, and a few showed non-linear effects, indicating possible compensatory mechanisms or context-dependent roles in survival. Overall, our CRISPR screen successfully identified and validated genes essential for cell survival under hypoxia, demonstrating high reliability.

### 2.5. Identification of Key Molecular Hubs in Ocular Disease Pathways Through Convergence of Hypoxia-Driven Transcriptomic and Genetic Dependency Profiles

#### 2.5.1. Transcriptomic Characterization of Hypoxia-Sensitive Genes Identified by CRISPR Screening

**Figure 6:**
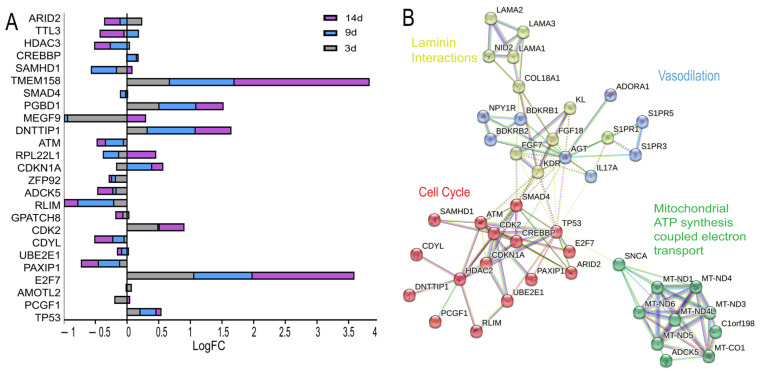
Transcriptomic profiling and network integration of hypoxia-essential genes in ARPE-19 cells. (**A**). Transcriptional dynamics of hypoxia-essential genes. Bar plots showing RNA-seq-derived expression levels of hypoxia-essential genes across different time points under 1% O_2_. Bars are color-coded by time point: gray for 3 days, blue for 9 days, and purple for 14 days, with expression compared to normoxia (21% O_2_). Genes shown exhibit significant transcriptional changes (log_2_ fold change), suggesting dynamic regulation at the RNA level during chronic hypoxic adaptation. (**B**). Functional network integration of transcriptomic and CRISPR screening data using STRING. Protein–protein interaction network illustrating hypoxia-essential genes mapped from the CRISPR screen and RNA-seq dataset. Nodes are color-coded by functionally annotated clusters based on STRING enrichment: mitochondrial function (green), cell cycle regulation (red), laminin interactions (yellow), vasodilation (purple). Some essential genes identified by CRISPR screening that do not fall into any enriched functional category, indicating novel or poorly characterized roles in hypoxia adaptation. Edges represent known or predicted functional interactions based on STRING confidence scores.

To gain mechanistic insight into genes identified as essential under hypoxia, we analyzed their transcriptional profiles using RNA-seq data (Figure 6A). This approach enabled the evaluation of how CRISPR screen hits were transcriptionally regulated under 1% hypoxia, offering an integrated view of gene essentiality and expression dynamics.

The RNA-seq analysis revealed distinct expression patterns among sensitivity genes over time. Some genes, including *ARID2*, *HDAC2*, and *UBE2E1*, exhibited early upregulation followed by downregulation, suggesting roles in acute stress response. Others, such as *TTLL3* and *GPATCH8*, displayed a down–up–down pattern indicative of a staged adaptation. A subset of genes, including *MEGF9*, *RPL22L1*, *CDKN1A*, and *PCGF1*, showed delayed transcriptional upregulation, while a separate group including *CREBBP*, *TMEM158*, *PGBD1*, *DNTTIP1*, *CDK2*, *E2F7*, *AMOTL2*, and *TP53* remained consistently upregulated. In contrast, *SMAD4*, *ATM*, *ZFP92*, *ADCK5*, *RLIM*, *CDYL*, and *PAXIP1* showed persistent downregulation despite their essentiality. Interestingly, some CRISPR screen hits such as *IL17A*, *LCE5A*, and *C16orf78* did not exhibit differential expression in RNA-seq data, suggesting potential regulatory mechanisms beyond transcription.

#### 2.5.2. Network Analysis of RNA Seq Mediated Key Hypoxia Functional Nodes and Cell Fitness

We used STRING network analysis (v12.0; STRING Consortium, Zurich, Switzerland) to integrate hypoxia-driven transcriptomic data (Figure 2D) with essential genes from our CRISPR screen revealing key molecular hubs critical for retinal cell survival under low oxygen (Figure 6B), many of which are relevant to AMD and other ocular diseases. From hypoxia-responsive transcriptomic modules, we prioritized the three most interconnected and enriched hubs for integration. Overlaying CRISPR hits onto these hubs pinpointed essential viability genes aligned with dominant hypoxia-adaptive pathways, highlighting candidate regulators of RPE dysfunction and AMD pathogenesis.

Several functional modules emerged as key players in the cellular response to hypoxia and cell survival. Small GTPase signaling, including genes like *RHOQ* and *RAC* family members, suggests a role in cytoskeletal dynamics and cell behavior relevant to retinal health. Another module, involved in inflammatory signaling and stress responses (e.g., *S1PR* and *ADORA* family members), highlights a link between hypoxia and these crucial processes in the retina. Cell cycle regulation genes indicate that hypoxia impacts cell proliferation, a process relevant to retinal dysfunction. Finally, the prominent cluster of mitochondrial genes underscores the critical role of energy metabolism in the cellular response to hypoxia and retinal stress.

Together, these modules form distinct hypoxia-adaptive networks. This intersection highlights key hubs in the cellular response to the hypoxic microenvironment common in ocular diseases, potentially mediating both immediate transcriptional adaptation and long-term survival strategies in retinal cells. Additionally, these networks point to core regulators of retinal cell viability under hypoxic stress.

## 3. Discussion

Our findings build upon and extend prior acute hypoxia studies by systematically modeling sustained low-oxygen exposure over extended timeframes. Through an integrated analysis of transcriptomic and genetic dependency data, we uncover critical oxygen-sensitive transcriptional modules and functionally essential genes that converge on key pathways, offering a comprehensive framework for understanding how long-term hypoxia may contribute to RPE dysfunction in aging and retinal disorders.

Retinal oxygen tension in vivo typically ranges from ~1–5% O_2_ and varies substantially by retinal layer, age, metabolic demand, and disease state. This spatial and temporal heterogeneity is a defining feature of the outer retina, where the RPE cells reside, becoming increasingly dysregulated during retinal aging and degeneration. Accordingly, we selected oxygen levels that model both physiological and pathological hypoxic states relevant to RPE biology. Exposure to 5% O_2_ approximates a low but generally physiological outer retinal environment, whereas 1% O_2_ represents a widely used model of severe hypoxia associated with advanced AMD, geographic atrophy, and ischemic retinal disease [25,26]. Consistent with prior studies demonstrating pathological stress responses in RPE at ~1% O_2_ [27], we used 1% O_2_ specifically to model disease-relevant hypoxia rather than normal physiology.

Importantly, the dominance of temporal effects in PCA underscores that hypoxic adaptation in RPE cells is a progressive process rather than a static response to oxygen tension. Early hypoxia engages rapid stress signaling and compensatory transcriptional programs that support short-term survival, whereas prolonged exposure leads to stable rewiring of metabolic, structural, and regulatory pathways reflective of chronic stress states relevant to retinal degeneration. This temporal stratification is particularly important in the retina, where chronic hypoxic stress accumulates over years rather than hours or days, and thus long-term adaptation more closely reflects disease-relevant biology than acute exposure models. To enable systematic, genome-wide interrogation of hypoxia-dependent genetic vulnerabilities over extended culture durations, we employed ARPE-19 cells for CRISPR loss-of-function screening. Large-scale functional genomics studies require robust proliferation, high transduction efficiency, and sustained viability, which remain technically challenging in primary and iPSC-derived RPE due to limited expandability and inter-sample variability [28]. Although ARPE-19 is an immortalized model, it retains key stress- and hypoxia-responsive features relevant to RPE biology [25]. To minimize passage-associated phenotypic drift, all experiments were restricted to passages 22–28, consistent with prior reports [29].

We began our investigation by characterizing how different oxygen levels elicit distinct and temporally dynamic transcriptional programs. Chronic exposure to severe hypoxia (1% O_2_) induced a stress-adapted, proliferative phenotype marked by the upregulation of *E2F* targets, G2/M checkpoint regulators, and DNA repair machinery consistent with hypoxia-driven cell cycle adaptation and genomic instability reported in epithelial cancers [30,31,32]. Hypoxia is well known to impair DNA replication fidelity and induce DNA damage, activating checkpoint pathways that, if unresolved, drive apoptosis or permanent cell-cycle arrest. Thus, survival under severe hypoxia requires tight coordination of cell-cycle progression and genome maintenance mechanisms. Such processes may also contribute to pathological RPE proliferation observed in proliferative vitreoretinopathy and neovascular AMD [33].

In contrast, mild hypoxia (5% O_2_) activated *TNF-α*, *NF-κB*, and type I interferon signaling, reflective of a parainflammatory phenotype. These pathways are associated with immune modulation during ischemia and neurodegeneration [34,35,36], and closely resemble the chronic low-grade inflammation observed in early AMD stages, where altered RPE–choroid immune communication contributes to disease onset [33]. These findings support the notion that spatially distinct hypoxic zones within the retina may recapitulate specific AMD pathologies [37]. These findings support a model in which moderate, spatially restricted hypoxia may promote inflammatory signaling without overt loss of viability, whereas more severe hypoxia crosses a threshold into pathological stress and cellular dependency. Because our detailed downstream analyses focused on the 1% O_2_ condition, we did not systematically interrogate oxidative stress or antioxidant gene regulation under 5% O_2_, and therefore do not draw conclusions regarding redox responses to mild hypoxia. Future studies will be required to evaluate antioxidant and redox-regulatory pathways, including *SOD2*, *CAT*, and *GPX4*, under these conditions. To facilitate such analyses, the 5% O_2_ transcriptomic dataset will be deposited and made publicly available.

To further dissect hypoxia-induced functional changes, we examined core metabolic modules contributing to RPE vulnerability under chronic hypoxic stress. We observed enrichment of glycolytic pathways and suppression of mitochondrial oxidative programs, consistent with a shift from OXPHOS to glycolysis. This transcriptional shift is indicative of metabolic reprogramming, as previously reported in RPE cells and retinal tissues [4]. This inferred metabolic transition at 1% O_2_ aligns with extensive evidence demonstrating hypoxia-driven constraints on mitochondrial respiration and compensatory glycolytic ATP production in hypoxia-sensitive tissues [38,39]. While we did not directly assess metabolic flux, these transcriptional changes align with established hypoxia-induced adaptations that sustain ATP production, limit ROS generation, and mitigate oxidative stress under low-oxygen conditions [14,15]. In the RPE, which utilizes a hybrid metabolic program, this reprogramming may initially support survival but later leads to metabolic imbalance. Enhanced glycolysis and impaired mitochondrial oxidation disturb NAD^+^/NADH balance, lipid metabolism, and redox homeostasis, factors known to promote RPE stress and photoreceptor dysfunction in AMD [40,41]. Within this metabolic context, our screen identified *ADCK5* as a hypoxia-essential gene. While not well characterized in the retina, *ADCK5* is a predicted mitochondrial kinase within the aarF domain–containing family, consistent with mitochondrial adaptation being critical for hypoxic survival. STRING network analysis places *ADCK5* within mitochondrial and metabolic modules, supporting its functional relevance despite limited transcriptional induction. Analogous family members such as *ADCK3* (*COQ8A*) regulate coenzyme Q biosynthesis and electron transport [42], suggesting that loss of *ADCK5* may impair mitochondrial function, mitophagy, or respiratory efficiency, rendering RPE cells vulnerable under hypoxic stress. Given the high energy demands of the RPE, maintaining mitochondrial homeostasis is vital for long-term retinal integrity [37,43,44].

In parallel, hypoxia affected vasoregulatory and neuroimmune modules. A notable finding from our CRISPR screen was the identification of *IL17A* as a hypoxia survival gene. Although traditionally linked to Th17-driven inflammation and ocular diseases such as diabetic retinopathy, *IL17A* has also been implicated in epithelial stress responses and tissue repair [45,46,47]. Its expression has been documented in the human retina and RPE/choroid, with elevated levels reported in AMD [46,48]. Functional loss sensitized ARPE-19 cells to hypoxia-induced death, suggesting a protective, cell-autonomous role under stress conditions. These results highlight a previously underappreciated contribution of *IL17A* signaling to hypoxia resilience in the retina. Simultaneously, repression of the Laminin Interaction module under chronic 1% hypoxia points to early disruption of ECM and Bruch’s membrane components, mirroring structural alterations observed in AMD [16,17,33,49].

Our CRISPR screen further revealed that chronic hypoxia reshapes cell cycle regulation in RPE cells. Key regulators including *TP53*, *CDKN1A* (*p21*), *E2F7*, and *CDK2* were essential for survival. *TP53* activation under hypoxia promotes antioxidant defenses and DNA repair, and its loss sensitizes cells to hypoxic stress [50]. *CDKN1A*, a *TP53* target, enforces G1/S arrest to limit replication stress, a protective mechanism in retinal and neural injury [51]. Beyond these canonical factors, we identified a broader survival module encompassing *ATM*, *SMAD4*, *HDAC3*, *PCGF1*, and *ARID2* that lacked corresponding transcriptional induction. This illustrates a key distinction between functional and transcriptomic readouts: CRISPR screens capture protein- and pathway-level dependencies that may arise through post-transcriptional, epigenetic, or signaling mechanisms rather than changes in steady-state mRNA abundance [23,52]. Under hypoxia, regulators such as *SMAD4* and *HDAC3* can drive chromatin and signaling adaptations without marked transcriptional changes, resulting in transcript-independent essentiality. Dysregulation of these genes has been linked to retinal degeneration, with *TP53* and *CDKN1A* contributing to RPE senescence in AMD [53]; defective *ATM* worsens retinal inflammation [54]; and *HDAC2* mediates oxidative stress and aging-related changes in the retina [55]. Several genes identified in our screen lack prior functional annotation in RPE or AMD biology, highlighting them as compelling novel hits. *GPATCH8*, linked to RNA processing and spliceosome-associated functions, may contribute to maintaining transcript fidelity under hypoxic stress, a critical requirement during prolonged low-oxygen exposure. Together with *ADCK5*, these genes expand the repertoire of hypoxia-essential regulators and underscore the importance of RNA metabolism and mitochondrial integrity in sustaining RPE viability under chronic stress.

Our findings align with and expand on recent studies. For example, Jain et al. [22] showed that Complex I inhibition improved hypoxic survival in K562 cells, suggesting context-dependent benefits of limiting OXPHOS. In contrast, our data indicate that mitochondrial integrity is essential in ARPE-19 cells, highlighting cell type-specific hypoxic dependencies. Wang et al. [24] reported that mild hypoxia promotes ARPE-19 proliferation, whereas prolonged exposure induces mitochondrial ROS and apoptosis, consistent with our temporal framework. Kurihara et al. [4] demonstrated that *Vhl* deletion in RPE drives metabolic dysfunction and photoreceptor degeneration through HIF-mediated reprogramming. Additional studies show that hypoxic RPE secretes angiogenic factors such as bFGF, promoting endothelial proliferation [56]. These context-specific metabolic and cell cycle states shape retinal disease trajectories. In dry AMD, RPE senescence and arrest impair photoreceptor support [53,57], whereas in wet AMD and PVR, hypoxia promotes aberrant RPE proliferation and angiogenesis [58,59]. Therapeutic efficacy may therefore depend on the hypoxia-imposed cell cycle state: hypoxia-arrested or senescent RPE cells may resist conventional cytotoxics but respond preferentially to senolytic or metabolic interventions [60,61]. In dry AMD, strategies aimed at restoring controlled cell cycle re-entry or enhancing metabolic resilience, such as nicotinamide supplementation [61], may be beneficial. In contrast, proliferative conditions may require inhibition of cell cycle drivers or upstream regulators including *VEGF*, *mTOR*, and *HIF1A* [5,62], with additional opportunities in *Wnt/β-catenin* and *Notch* signaling pathways [63,64]. Although reliance on a single immortalized RPE cell line is a limitation, ARPE-19 cells enable scalable genome-wide CRISPR screening that remains challenging in primary or iPSC-derived RPE systems, rendering these findings discovery-focused. RNA-seq was performed at day 14, whereas CRISPR screening extended to day 28, reflecting the distinct biological questions addressed by transcriptomic versus functional assays. Together, these approaches capture both hypoxia-induced transcriptional adaptation and long-term survival dependencies, which will be validated in primary, iPSC-derived, and in vivo RPE models in future studies.

Taken together, these results demonstrate that RPE adaptation to chronic hypoxia is multifaceted, involving coordinated remodeling of metabolic, genomic, structural, immune, and cell cycle pathways that collectively determine cellular fitness and influence retinal disease progression.

## 4. Materials and Methods

### 4.1. Lead Contact

Inquiries regarding resources and reagents should be directed to the lead contact, who will fulfill such requests.

### 4.2. Experimental Model Details

Cell culture of ARPE-19

Human ARPE-19 cells (CRL-2302; ATCC, Manassas, VA, USA) were cultured in DMEM (Thermo Fisher Scientific, Waltham, MA, USA) supplemented with 10% fetal bovine serum (FBS, Gibco; Thermo Fisher Scientific, Waltham, MA, USA) at 37 °C with 5% CO_2_ [65]. Assays were performed with ARPE-19 cultures between passages 22 and 28 [29].

Cas9-stable cell line generation

A Cas9-stable cell line was generated by lentiviral transduction. Parental cell line was transduced with a Cas9 and blasticidin-resistance construct in 12-well plates (Corning Inc., Corning, NY, USA), centrifuged at 1000× *g* for 2 h in the presence of 8 μg/mL polybrene (Sigma-Aldrich, St. Louis, MO, USA). Following overnight incubation at 37 °C with 5% CO_2_, cells were selected with 20 μg/mL blasticidin (Gibco; Thermo Fisher Scientific, Waltham, MA, USA). ARPE-19 Cas9 activity was measured by BD LSRFortessa flow cytometer (BD Biosciences, San Jose, CA, USA) 9 days post-selection.

Virus titration

To determine the optimal viral dose (MOI) for a CRISPR Brunello genome-wide screen, a virus titration was performed in duplicate. Two million Cas9-expressing RPE cells were counted and seeded in triplicate in 12-well plates and transduced with 100 μL of serially diluted Brunello sgRNA library lentivirus (Broad Institute, Cambridge, MA, USA), including a no-virus control (also in triplicate). Transduction was enhanced by spinfection (1000× *g* for 1 h) using 1 mL of media containing 8 μg/mL polybrene. After spinfection, cells were transferred to T75 flasks (Corning Inc., Corning, NY, USA) (4 million cells in 4 mL media, scaled up for a Corning 2-chamber CellSTACK flasks (Corning Inc., Corning, NY, USA) in the actual screen and incubated at 37 °C with 5% CO_2_ and 21% O_2_. The following day, the media were replaced. Two days post-transduction, 4 μg/mL puromycin was added to each serially diluted well, along with no-puromycin controls. To determine transduction efficiency, cells were allowed to undergo puromycin selection until uninfected cells were eliminated. Then, the remaining cells in each well (both with and without puromycin) were collected and counted. The MOI was calculated by comparing cell counts from puromycin-selected (+) and unselected (−) wells for each viral dilution. The dilution corresponding to an MOI of 0.3 was selected for the screen.

Genome-wide CRISPR screening

Approximately 1.9 billion ARPE-19 Cas9 cells were infected with the Brunello sgRNA library (Broad Institute, Brunello_XPR003 M-AF13-20211005) at a MOI of 0.3, resulting in roughly 500 infected cells per sgRNA. Two million cells per well were transduced via spinfection in 12-well plates, using 1 mL of media containing 8 μg/mL polybrene at 1000× *g* for 1 h. Twelve 12-well plates were used per biological replicate. Following spinfection, cells were transferred to four 2-chamber Corning CellSTACKs at a density of approximately 65 million cells per stack and incubated at 37 °C with 5% CO_2_ and 21% O_2_. This process was performed for three biological replicates. Fresh media were added the day after transduction, and selection with 4 μg/mL puromycin in DMEM began two days post-transduction. Five days post-infection, an in-line assay was performed, and cells were detached by trypsinization and counted. For each replicate, 40 million cells (approximately 500-fold library coverage) were harvested and seeded into three 5-chamber Corning CellSTACKs containing 800 mL of fresh medium supplemented with 20 μg/mL blasticidin and 4 μg/mL puromycin per condition. These CellSTACKs were then placed in tissue culture incubators, each set to a different oxygen tension: 21%, 5%, and 1% O_2_. The specified oxygen tension in each incubator was maintained via pulsed nitrogen gas (N_2_) regulated by a feedback loop. Cells were passaged on days 0, 9, 14, and 28 for each oxygen condition and the replicates, with a seeding density of 40 million at the start of each passage. All tissue culture handling was performed under normoxic conditions (21% O_2_). After more than 10 population doublings, 40 million cells per replicate for each condition were pelleted, and genomic DNA was extracted from the frozen pellets using the Machery-Nagel NucleoSpin Blood L Midi kit (Macherey-Nagel, Düren, Germany; distributed by Takara Bio, San Jose, CA, USA) according to the manufacturer’s instructions. To achieve >500× coverage of the Brunello library (assuming 6 μg of genomic DNA per 1 million cells), 24 separate 100 μL PCR reactions were performed with 10 μg of genomic DNA in each reaction, using Titanium Taq DNA Polymerase (Clontech; Takara Bio, San Jose, CA, USA). The sgRNA sequences were amplified using a mix of eight P5 primers (ARGON) with varying-length stagger regions and a unique P7 primer (to barcode each reaction), resulting in amplicon sizes of 250–550 bp. The resulting amplicons were then combined and purified using AMPure beads (Beckman Coulter, Brea, CA, USA) according to the manufacturer’s instructions. The samples were quantified by 4200 TapeStation (Agilent Technologies, Santa Clara, CA, USA), then mixed, and sequenced on a NextSeq 500 system (Illumina, San Diego, CA, USA) using 75 bp single-end sequencing.

Processing and analysis of sgRNA sequencing data

sgRNA sequencing data analysis involved several steps. Raw sequencing reads were demultiplexed using bcl2fastq and quality filtered, considering mapping percentage and PCA to remove low-quality sequences. SgRNA quantification across all samples was performed using custom Perl scripts, which extracted sgRNA sequences from raw fastq files either by specifying read locations or identifying flanking primer sequences for variable sgRNA positions. Extracted sequences were matched to the Brunello sgRNA library, retaining only perfectly aligned reads to generate the count matrix. Downstream analyses were conducted using R scripts (v4.3.1; R Foundation for Statistical Computing, Vienna, Austria) for quality control, including assessments of mapping efficiency, library representation, replicate correlation, and sample variability via Principal Component Analysis (PCA). Read counts were normalized using the TMM (trimmed mean of M-values) method implemented in the edgeR package (v4.0.16; Bioconductor, Fred Hutchinson Cancer Center, Seattle, WA, USA). Differential sgRNA representation between experimental conditions was determined using limma package (v3.58.1; Bioconductor, Fred Hutchinson Cancer Center, Seattle, WA, USA), with enrichment scores calculated based on the experimental design and contrast matrix. Finally, rank aggregation analysis (RRA) (RobustRankAggreg package v1.1; R Foundation for Statistical Computing, Vienna, Austria) was used to identify statistically significant gene-level enrichments, highlighting sgRNAs that were significantly enriched or depleted based on [adjusted *p*-value threshold, fold change cutoff].

RNA extraction, sequencing, and expression analysis

ARPE-19 cells were cultured under 21%, 5%, and 1% oxygen conditions for 3, 9, and 14 days. For each treatment group and time point, three biological replicates were collected, with 1 million cells per replicate. Total RNA was extracted from a million cells using the Zymo-Spin IIC™ column (Zymo Research, Irvine, CA, USA). Briefly, cell pellets were homogenized in Trizol (Invitrogen; Thermo Fisher Scientific, Waltham, MA, USA), followed by ethanol addition and homogenization. The solution was loaded onto a Zymo-Spin IIC™ column and centrifuged. After discarding the flow-through, the column was transferred, and RNA was treated with DNase. Following two prewash steps and centrifugation, RNA wash buffer was added and centrifuged. The column was transferred to an RNase-free tube, and RNA was eluted with DEPC-treated nuclease-free water via centrifugation. RNA sequencing libraries were prepared using the NEBNext^®^ Ultra II Directional RNA Library Prep Kit (New England Biolabs, Ipswich, MA, USA; distributed by Illumina, San Diego, CA, USA) according to the manufacturer’s instructions. RNA purity was assessed spectrophotometrically NanoDrop One spectrophotometer (Thermo Fisher Scientific, Waltham, MA, USA), with concentrations estimated between 500 and 2000 ng/μL. Next-generation sequencing (NGS) was performed on 27 total RNA samples, generating 75 bp paired-end reads at a sequencing depth of 50 million reads per sample. RNA-seq data analysis included read alignment, transcript quantification, and differential expression analysis. Raw sequencing data were generated in FASTQ format, and quality control of both raw and trimmed reads was performed using FastQC, with results summarized by MultiQC (v1.14; Phil Ewels, Stockholm, Sweden). Adapter trimming and quality filtering were conducted using Trim Galore (v0.6.10; Babraham Institute, Cambridge, UK). Reads were aligned to the human reference genome (Ensembl Release 84) using the STAR aligner, and transcript abundance was quantified with FeatureCounts. Gene-level expression values were obtained from transcript-level counts using the RSubread R package (v2.16.0; Bioconductor, Fred Hutchinson Cancer Center, Seattle, WA, USA). Differential expression analysis was performed using the Limma-Voom package (v3.58.1; Bioconductor, Fred Hutchinson Cancer Center, Seattle, WA, USA). The RNA-seq workflow was executed using the RNAseq CWL pipeline within the ARTEMIS back-end system. RNA-seq quality was assessed using MultiQC, confirming high data integrity.

Gene ontology and pathway analysis

Ingenuity Pathway Analysis (IPA; v23.0; QIAGEN, Redwood City, CA, USA) was used to identify enriched biological pathways or processes associated with the differentially expressed genes identified for each treatment group and time point as well as the observed sgRNA changes. Fisher’s exact test was employed within IPA to calculate *p*-values, assessing the likelihood that the observed association between the dataset and each assigned biological function occurred by random chance.

Cell viability assay for target validation

To knock out the top 30 CRISPR targets along with the non-targeting vector, ARPE-19 Cas9-expressing cells were transduced with lentiviral vectors encoding specific gene-targeting sgRNAs (high MOI) in the presence of 8 μg/mL polybrene. Puromycin selection (4 μg/mL) began 24 h post-transduction and was maintained for 14 days to establish a stable transduced cell population.

Following stable cell line generation, cells (all gene knockouts and the non-targeting control) were plated in 96-well plates in triplicate for time-course analysis at 3, 9, 14, and 28 days under three oxygen tensions: normoxia (21% O_2_), 1% hypoxia, and 5% hypoxia. Seeding density was assessed the day after plating using the Incucyte ZOOM live cell imaging system (Essen Bioscience, Ann Arbor, MI, USA) to account for initial variations in cell number. The top 15 target genes and the non-targeting control were seeded on one plate, and the remaining 15 genes were seeded on a separate plate under identical conditions. The recorded seeding densities were used to calculate normalization ratios between normoxia and hypoxia groups for each gene, correcting for confluency differences in subsequent data analysis. At each time point, cell viability was assessed using the CellTiter-Glo^®^ Luminescent Cell Viability Assay (Promega, Madison, WI, USA) according to the manufacturer’s instructions. Briefly, equilibrated and reconstituted CellTiter-Glo^®^ reagent (100 μL) was added to each well, including blank and untreated control wells. Plates were gently mixed for 2 min and incubated at room temperature for 10 min to stabilize the luminescent signal before measuring luminescence using a plate reader.

For data analysis, background luminescence was subtracted from raw CellTiter-Glo (CTG) values. The previously calculated seeding density ratios were then applied to the normoxia and hypoxia group CTG values at each time point to correct for seeding variability. Finally, hypoxia values were normalized to their respective normoxia controls for each gene, and these normalized values were compared to the non-targeting control to determine the effect of each gene knockout under hypoxic conditions.

## Data Availability

The original data presented in the study are openly available at https://www.ncbi.nlm.nih.gov/ (access on 17 December 2025).

## Supplementary Materials

The following supporting information can be downloaded at: https://www.mdpi.com/article/10.3390/ijms27062857/s1.
